# Antipsychotic medication non-adherence among schizophrenia patients in Central Ethiopia

**DOI:** 10.4102/sajpsychiatry.v24i0.1124

**Published:** 2018-03-05

**Authors:** Minale Tareke, Siranesh Tesfaye, Desalegn Amare, Tilahun Belete, Andargie Abate

**Affiliations:** 1 College of Medicine and Health Science, Bahir Dar University, Ethiopia; 2 Department of Psychiatry, Felege Hiwot Referral Hospital, Ethiopia; 3 College of Health Science, Psychiatry Unit, Department of Nursing, Mekelle University, Ethiopia

## Abstract

**Background:**

Despite the fact that adherence to antipsychotic medications is the cornerstone in the treatment and prevention of relapse of the disease, non-adherence is a major problem among schizophrenia patients. The purpose of this study was to assess the magnitude and factors associated with antipsychotic medication non-adherence among schizophrenia patients in Amanuel Mental Specialized Hospital.

**Method:**

An institution-based cross-sectional study was conducted among 412 people with schizophrenia at Amanuel Mental Specialized Hospital from April to May 2014. Non-adherence was assessed using the questionnaire of Morisky medication adherence rating scale and semi-structured questions for assessment of associated factors. Logistic regression analysis was used to assess predictors of non-adherence.

**Results:**

Prevalence of non-adherence was 41.0% among schizophrenia patients. Living in rural areas (adjusted odds ratio [AOR] = 2.07; 95% confidence interval [CI]: 1.31, 3.28), current substance use (AOR = 1.67; 95% CI: 1.09, 2.56), long duration of treatment (AOR = 2.07; 95% CI: 1.22, 3.50) and polypharmacy (AOR = 2.13; 95% CI: 1.34, 3.40) were found to be significantly associated with non-adherence.

**Conclusion:**

The results indicate that non-adherence to antipsychotic medication was a major problem among patients with schizophrenia. Reducing the number of antipsychotic medications and availing drugs in rural areas may decrease the level of non-adherence.

## Introduction

Schizophrenia is a severe illness which affects all life aspects of the patients including work, self-care and capacity to establish interpersonal relationships.^1^ It is one of the top 10 causes of long-term disability in the world, affecting about 1.0% of the population.^2^

Antipsychotic drugs have become the cornerstone of treatment for schizophrenia. These are effective in reducing psychotic symptoms, preventing psychotic relapses and improving psychosocial functioning.^3^ However, non-adherence to medication is one of the biggest problems, increasing re-hospitalisation and persistent psychotic symptoms, and the most challenging aspect of treatment.^4,5,6^

Non-adherence can cause high rates of relapse within 5 years of recovery from the first episode.^7^ Thus, lack of adherence to pharmacological treatment is associated with worsening of symptoms, poor prognosis, high costs and unnecessary adjustments in the medical prescriptions.^8^

A report in Poland revealed that treatment adherence in the first month was very low, but application of telemedicine monitoring systems would improve the compliance in patients with the worst compliance.^9^ Many studies reported that the level of medication adherence for antipsychotic treatment was ranged from 20.0% to 70.0%.^10,11,12^

Different reports in Ethiopia have shown that non-adherence to antipsychotics medication was varied from 26.5% to 47.9%.^13,14,15,16^ Other qualitative studies showed that medication side effects, poverty, lack of family supports, duration of illness and stigma, substance use, alcohol consumption and smoking are some of the factors affecting the medication adherence of schizophrenia.^17^ Thus, non-adherence remains a challenge for patients with psychiatric disorders and their health care providers, contributing to a substantial worsening of disease, frequent relapse, increased mortality, re-hospitalisation and increased health care costs.^18^

The magnitude and consequences of non-adherence to antipsychotic drugs among schizophrenia patients vary from one place to another, suggesting the need of study in the local context. However, there is limited evidence in the study area. Therefore, this study was intended to fill the gap by assessing the magnitude and factors associated with antipsychotic medication non-adherence among people with schizophrenia at Amanuel Mental Specialized Hospital (AMSH).

## Methods

### Study setting and design

A cross-sectional study design was used at AMSH from April to May 2014. AMSH is one of the pioneer Ethiopian hospitals, established in 1938 by the Italians. The hospital is playing its pivotal role as a training institute for psychiatric professionals so as to expand the service throughout the country by introducing psychiatry services in the primary health care system of the country. Annually, about 51 204 schizophrenia patients visited the hospital for regular follow-up services at outpatient departments, and, on average, 4267 schizophrenia patients had monthly follow-ups. All schizophrenia patients who had regular follow-up at AMSH were taken as a source population, while those schizophrenia patients who were available during data collection were considered as a study population. Schizophrenia patients aged above or equal to 18 years, who had one or more previous visits and who were on follow-up during the study period at outpatient departments of AMSH were recruited for the study. However, patients with serious medical or neurological illnesses, who had no insight and who were unable to communicate were excluded from the study.

### Sample size determination and procedure

A single population proportion formula {[*Z*^2^*p* (1−*p*)]*/d*2} with an assumption of 95.0% confidence level, 5% margin of error and 50% proportion of schizophrenia patients with non-adherence to their medication(p) and 10% non-response rate was used to determine the final sample size of 423.

The total number of schizophrenia patients who visited the hospital over the previous 6 months was taken from records and the average number of patients per month was calculated and found to be 4267. Therefore, systematic random sampling technique was used to include every 10th (4267/423 = 10) schizophrenia patient available at the clinic during the data collection period.

### Data collection method and instrument

Data were collected using a semi-structured and pretested questionnaire by face-to-face interview technique. Chart review was used to collect the type and number of antipsychotic drugs and duration of medication. Two trained psychiatry nurses working in the mood disorder department of the hospital collected the data for a period of approximately 1 month. The questionnaire included socio-demographic data, Morisky medication adherence rating scale (MMARS)-8 items and semi-structured questions for assessment of associated factors. MMARS-8 is extensively used to measure non-adherence in Ethiopia.^19,20,21^ The cut-off point of 3 and above was used to define non-adherence in this study. The questionnaire was translated into Amharic and back to English by language experts and a psychiatrist for checking its consistency.

### Data quality assurance and analysis

Data were entered to Epi Info version 3.5 after checking for completeness and then exported to SPSS version 20 for further analysis. Descriptive statistics analysis was performed for variables and the results were presented using tables, percentage, mean and standard deviation. Bivariate logistic regression was first conducted to identify potential confounding factors and variables with a *p* < 0.2 were entered to multivariable logistic regression models using backward selection method to identify factors associated with non-adherence. An adjusted odds ratio with 95.0% confidence interval was used to interpret the strength and significance of the association.

## Ethical considerations

Ethical clearance was obtained from the ethical research review committee of the University of Gondar and Amanuel Mental Specialized Hospital. Written consent was obtained from each study participant during data collection. The purpose and procedure of the study were clearly explained to all the participants. The participants were assured that their identity would remain anonymous and their responses would be kept confidential. The right was given to participants to decide whether they wish to be part of the research or not. Furthermore, they were assured that they can withdraw from the study at any time and it would not have any effect on their next follow-up treatment.

## Results

### Socio-demographic characteristics of schizophrenia patients

Out of the total 423 recruited patients, 412 filled in the questionnaire completely, giving a response rate of 97.4%. The mean age of schizophrenia patients was 35.28 years with a standard deviation of 10.35 years. Most (68.0%) of the respondents were living in urban areas, single (65.5%) and orthodox religion followers (56.1%). About half of the respondents (47.6%) were jobless and nearly four in every five (73.3%) were earning less than 750 Ethiopian birr/month (less than US$33) (Table 1).

**TABLE 1:** Distribution of socio-demographic characteristics of schizophrenia patients attending at Amanuel Mental Specialized Hospital, Addis Ababa, Ethiopia, June 2014 (*n* = 412).

Variable	Frequency	%
**Sex**		
Male	287	69.7
Female	125	30.3
**Age**		
≤ 25	71	17.2
26–35	163	39.6
≥ 36	178	43.2
**Marital status**		
Single	270	65.5
Married	80	19.4
Divorced	32	7.8
Widowed	6	1.5
**Residence**		
Urban	280	68.0
Rural	132	32.0
**Ethnicity**		
Amhara	148	35.9
Oromo	112	27.2
Gurage	93	22.4
Tigre	21	5.1
Others	38	9.2
**Educational status**		
Uneducated	49	11.9
1–8 grades	126	30.6
9–12 grades	157	38.1
Diploma and above	80	19.4
**Occupation**		
Employed	68	16.5
Private business	70	17.0
Daily labourer	37	9.0
Jobless	196	47.6
Student	20	4.9
Housewife	21	5.1
**Monthly income (Ethiopian birr)**		
< 750 birr	302	73.3
750 birr–1199 birr	47	11.4
≥ 1200 birr	63	15.3

### Clinical and patient-related factors

Among the study participants, about half (48.1%) had been taking medication for fewer than 5 years. Nearly one-third (28.2%) of the participants were on antipsychotic polypharmacy (use of two or more antipsychotic drugs at a time). On the other hand, 29.6% of the participants had history of previous psychiatric admissions and history of substance use since initiation of treatment (39.3%) (Table 2).

**TABLE 2:** Distribution of clinical and patient-related factors among schizophrenia patients at Amanuel Mental Specialized Hospital, Addis Ababa, Ethiopia, June 2014 (*n* = 412).

Variable		Frequency	%
Duration of illness	< 3 years	78	18.9
	≥ 3 years	334	81.1
Antipsychotic	yes	116	28.2
polytherapy	No	296	71.8
Duration of treatment	< 2 years	111	26.9
	2–5 year	87	21.1
	> 5 years	214	51.9
Type of antipsychotics	FGA	250	60.7
	SGA	46	11.2
	FGA+FGA	96	23.3
	FGA+SGA	19	4.6
	FGA+FGA+SGA	1	0.2
Route of antipsychotics	Oral	368	89.3
	Injectable†	44	10.7
Number of admissions	None	290	70.4
	One	66	16.0
	≥ two	56	13.6
EPS	Yes	43	10.4
	No	369	89.6
Drug adherence	Yes	243	59.0
	No	169	41.0
Substance use	No	250	60.7
	Yes	162	39.3

EPS, Extrapyramidal side effects; FGA, first generation antipsychotics; SGA, second generation antipsychotics.

† , fluphenazine decanoate.

### Substances use among schizophrenia patients

**FIGURE 1:**
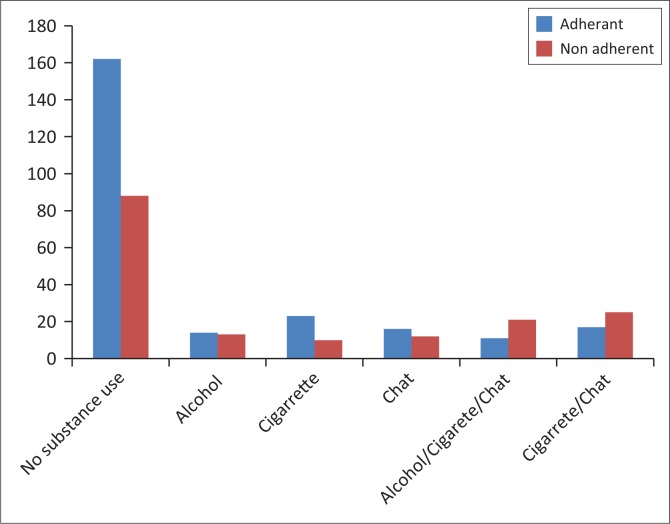
Bar chart showing the distribution of substance use among schizophrenia patients at Amanuel Mental Specialized Hospital, Addis Ababa, Ethiopia, June 2014 (*n* = 412).

Compared to alcohol use, current use of khat chewing and smoking cigarettes simultaneously were found to be high among non-adherent schizophrenia patients (Figure 1). Khat (chat) is one of the most common forms of drug use and abuse in many East African nations, especially in Ethiopia, involving chewing parts of the fresh green leaves. Chewing khat has stimulant and euphoric effects and is believed to have a relaxing effect, to increase concentration and is used to waste time.

### Factors associated with antipsychotic non-adherence

Prevalence of non-adherence was found to be 41.0% among schizophrenia patients in this study. Bivariate logistic regression analysis was performed to find the relationship of socio-demographic, patient and treatment-related variables with non-adherence of antipsychotics. The result of bivariate analysis revealed that place of residence, duration of illness, duration of treatment, number of psychiatric hospitalisation days, history of active substance use, only injectable antipsychotic use (fluphenazine decanoate) and antipsychotic polypharmacy were factors significantly associated with non-adherence. However, in multivariate logistic regression, place of residence, history of active substance use, duration of treatment and antipsychotic polypharmacy were found to be statistically significant.

Accordingly, patients who were living in rural areas were about twice as likely to be non-adherent to antipsychotic medication as compared to those living in urban areas (AOR = 2.07; 95% CI: 1.31, 3.28).

Regarding substance use, patients who were using psychoactive substances after initiation of treatment were nearly twice as likely to be non-adherent to antipsychotic medication compared to those who had no history of substance use (AOR = 1.67, 95% CI: 1.09, 2.56).

Moreover, participants who took medication for more than 5 years were twice as likely to be non-adherent to antipsychotic medication as compared to those who took for less than 5 years (AOR = 2.07, 95% CI: 1.22, 3.50).

Patients who were on antipsychotic polypharmacy were two times more likely to be non-adherent to their medication as compared to those who were on a single drug (AOR = 2.13, 95% CI: 1.34, 3.40) (Table 3).

**TABLE 3:** Factors associated with antipsychotic polypharmacy among schizophrenia outpatients under follow-up at Amanuel Mental Specialized Hospital, Addis Ababa, Ethiopia, June 2014 (*n* = 412).

Variable		Adherence		Odd ratio at 95% CI	
		Yes	No	Crude	Adjusted
Residence	Urban	178	102	1	1
	Rural	65	67	1.79 (1.18, 2.73)	2.07 (1.31, 3.28)*
Substance use (khat	No	162	88	1	1
Alcohol and tobacco)	Yes	81	81	1.84 (1.23, 2.75)	1.67 (1.09, 2.56)*
Duration of illness	< 5 years	95	48	1	1
	5–10 years	53	57	2.12 (1.27, 3.54)	1.75 (0.89, 3.47)
	> 10 years	95	64	1.33 (0.83, 2.13)	1.64( 0.66, 4.06)
Number of admissions	None	176	114	1	1
	One	40	26	1.00 (0.58, 1.73)	0.83 (0.46, 1.49)
	≥ Two	27	29	1.65 (0.93, 2.94)	1.24 (0.66, 2.35)
Duration of treatment	< 5 years	126	72	1	1
	5–10 years	42	53	2.20 (1.34, 3.63)	2.07 (1.22, 3.50)*
	> 10 years	75	44	1.02 (0.64, 1.64)	1.04 (0.62, 1.74)
Route of antipsychotics	Oral	212	156	1.75 (0.88, 3.46)	1.59 (0.77, 3.30)
	Injectable	31	13	1	1
Antipsychotics	No	191	105	1	1
polypharmacy	Yes	52	64	2.23(1.44, 3.46)	2.13(1.34, 3.40)*

1, Reference; CI, confidence interval.

* *p* < 0.05

## Discussion

Antipsychotic medication is the mainstay in the treatment of schizophrenia, and adherence to medication plays a pivotal role in controlling symptoms and preventing frequent relapses. From the finding of the present study, the magnitude of non-adherence among schizophrenia patients was found to be 41.0% (95% CI: 36.2%, 45.6%). Although it is clear that more than one factor is responsible for non-adherence among schizophrenia patients, place of residence, active substance use, duration of treatment and antipsychotic polypharmacy were the main factors identified in the current study.

This finding of this study was in line with the study conducted in Switzerland (44.8%).^22^ However, it is by far lower than the studies conducted in Egypt (74.0%),^11^ Jordan (64.2%),^23^ Nigeria (51.7% – 62.5%)^12,24^ and Bulgaria (55.8%).^25^ The possible explanation behind this might be the inclusion criteria. In Nigeria, comorbid psychoactive substance use and patients who had been on medication for less than 1-year were included, whereas schizophrenia patients who had more than one visit were included in our study. Furthermore, comorbid psychoactive substance use was investigated as an influencing factor for adherence in this study. The study conducted in Egypt also used a convenience sampling technique, suggesting the possible reason for the observed difference. In Bulgaria, 226 schizophrenia patients on long-term antipsychotic treatment were included, while the exclusion criteria were first episode of psychosis, substance dependence, date of the last hospitalisation less than 2 months prior to the study and change in type or dose of antipsychotic medication within the previous month. Furthermore, these discrepancies might be because of the difference in study participants, culture, time variation and settings.

The current estimate is larger than the result from studies conducted in France (30.0%),^26^ Jimma, Southwest Ethiopia (36.0%)^15^ and Mekelle, Northern Ethiopia (26.5%).^13^This might be because of the difference in study design, study setting, study year, socio-demographic data and type of screening tool used. The study performed in Mekelle had quite low rates of non-adherence that could be explained by differences in measuring medication adherence methods and sample size. The major difficulty in categorising the magnitude of non-adherence in schizophrenia patients is the lack of consistent and agreed criteria. For instance, the 10-item modified version of the Morisky Medication Adherence Scale (MMAS) was used in Mekelle, which was answered using ‘yes/no’ responses. A MMAS score equal to 3 or above indicated adherence, while less than or equal to 2 indicated non-adherence. However, in the current study, an 8-item MMAS with seven ‘yes/no’ questions and one question answered on a five-point Likert scale was used. In addition, the sample size variation and inclusion criteria might account for this difference.

A number of factors were significantly associated with non-adherence to antipsychotic medication. In this study, schizophrenia patients living in rural areas were found to be non-adherent compared to urban residents, which is supported by another study.^27^ The possible explanation might be related to long distance to health facilities, financial constraints to purchase medication, lack of services for mental health care and low community awareness regarding treatment of mental illness, which are more common in rural areas.

In addition, schizophrenia patients chewing chat, drinking alcohol and smoking cigarettes were non-adherent to their medications, which is in line with different studies.^4,17^ There was a significant negative relationship between substance abuse and non-adherence.^28^ Patients non-adherent to their medications were found to have higher percentage of present or past substance abuse.^29^

Furthermore, long duration of treatment and use of two or more antipsychotic drugs at a time were related to non-adherence, which was supported by a study conducted in Nigeria.^12^

The present study has some limitations. It was cross-sectional; therefore, temporal relationship between risk factors and non-adherence cannot be determined. Although we used psychiatry nurses working in the mood disorder department for data collection within the same hospital, it might have some information and recall biases. The patients’ report method used to measure treatment non-adherence might underestimate its magnitude compared to plasma concentrations.

## Conclusion

The aim of this study was to assess the prevalence and main factors associated with non-adherence. Thus, adherence to antipsychotics medication among patients with schizophrenia was a major problem in this study. Living in rural areas, substance use, long duration of treatment and taking more than one antipsychotic medication were the most important factors for increased non-adherence. Reducing the number of antipsychotic medications and availing drugs in rural areas may decrease the level of non-adherence. Furthermore, clinicians should emphasise the importance of screening of patients for substance abuse and offer brief motivational interventions for those in need of addiction rehabilitation services, which might lead to better adherence to their medications.
